# Correlation between chemosensitivity to anticancer drugs and telomerase reverse transcriptase mRNA expression in gastric cancer

**DOI:** 10.1186/1746-1596-8-33

**Published:** 2013-02-22

**Authors:** Lin Wang, Pei-Feng Li, Ming Geng, Yong-Cheng Cao, Ying-Chun Yin

**Affiliations:** 1 Department of Pathology, General Hospital of Jinan Military Command, 250031, Jinan, Shandong Province, China

**Keywords:** Gastric cancer, Anticancer drugs, Chemosensitivity, hTERT mRNA, MTT assay

## Abstract

**Background:**

The determination of sensitive chemotherapy drugs for gastric cancer (GC) is one of the greatest challenges of adjuvant therapy. Here we evaluated the chemosensitivity of GC to anticancer drugs and the telomerase reverse transcriptase (hTERT) mRNA expression, and investigated the relationship of them.

**Methods:**

The GC cells which were collected from 68 patients with primary GC were primary cultured. The chemosensitivity of GC cells to anticancer drugs was evaluated successfully using the MTT assay for 60 cases of GC cells, and the hTERT mRNA expression was examined in 60 cases of GC tissues and corresponding normal gastric mucosa and 6 cases of chronic superficial gastritis mucosa by in situ hybridization.

**Results:**

Taxol, Cisplatin and 5-Fluorouracil were in general more effective than Adriamycin and Mitomycin for GC cells, and the chemosensitivity to anticancer drugs was associated with tumor histological types and a worse tumor grade. Compared to normal gastric mucosa tissues, hTERT mRNA expression was significantly increased in GC (*P*<0.05), which was related with a worse differentiation and drug-resistance to 5-Fluorouracil or Adriamycin in GC.

**Conclusions:**

These data demonstrate for the first time that examinations of hTERT mRNA expression as an important factor could be used to select the chemotherapeutic drugs for GC patients.

**Virtual slides:**

The virtual slide(s) for this article can be found here: http://www.diagnosticpathology.diagnomx.eu/vs/1793217009875483

## Introduction

Although the incidence of gastric cancer (GC) has been significantly reduced in developed coutries, GC remains one of the most common malignancies worldwide and ranks second in terms of global cancer-related mortality [1,2]. In recent decades, chemotherapy plays an important role in the treatment of patients with advanced GC, although radical surgery is the only curative treatment. Chemotherapeutic regimens for cancer patients usually are based on the statistical results of clinical trials or the histological type of tumor rather than the cellular sensitivity to each anticancer drug [3-5]. Therefore, evaluation of the effectiveness of an anticancer drug prior to treatment is critical for determining the clinical efficacy of chemotherapy, avoiding adverse effects, and predicting the prognosis. The 3-(4,5-dimethylthiazol-2-yl)-2,5-diphenyl tetrazolium bromide (MTT) chemosensitivity test (MTT assay) is a rapid and quantitative method to evaluate the chemosensitivity of tumor cells to anticancer drugs [6-8].

Telomeres at the termini of each chromosome, which consist of TTAGGG repetitive DNA sequences and various binding proteins, have an important role in regulating the life-span of human cells and are synthesized by telomerase as a RNA-dependent DNA polymerase [9-11]. The core telomerase enzyme consists of two key components, the catalytic unit telomerase reverse transcriptase (hTERT) and the RNA template (hTER) [9,12]. Most of normal human somatic cells lack telomerase activity due to the stringent transcriptional repression of the hTERT gene. They thereby progressively lose their telomeres with each cell division and are eventually triggered to undergo senescence (irreversible cell growth arrest) when their telomeres become extremely short [13,14]. Telomerase activity or hTERT expression is detectable in up to 90% of human cancers, in contrast to its absence in normal tissues/cells except germ cells of the ovary and testis [15,16]. Therefore, assessment of hTERT expression is a useful diagnostic and prognostic marker in many types of human malignancies, including GC [11,17-20]. Furthermore, telomerase activity has been reported to be related to drug resistance in several cell lines [21,22]. Thus, hTERT expression could be used to evaluate the efficacy of anticancer drugs.

In this study, we evaluated the chemosensitivity to anticancer drugs and the hTERT mRNA expression in GC, and investigated the relationship between anticancer drug resistance and hTERT mRNA expression.

## Materials and methods

### Clinical data

The study included 68 patients with primary GC (mean age 55 years; range 33–75 years; male: female, 40:28) and was approved by the local ethics committee. The patients underwent gastrectomy at the General Hospital of Jinan Military Command from 2007 Jan to 2008 Dec. After surgery, the tumor specimens and distant normal gastric mucosa tissues were collected for this study. None of the patients enrolled in the study had received chemotherapy or radiotherapy prior to surgery, and there was no evidence of any other malignancies. The diagnoses of all GCs were histopathologically confirmed by examination of surgical specimens. The clinical stage of the patients and the pathologic grade of tumors were determined according to the TNM classification and WHO criteria, respectively. The stage was IA in 4 (5.9%), IB in 14 (20.6%), II in 23 (33.8%), IIIA in 15 (22.1%), IIIB in 6 (8.8%), IV in 6 (8.8%). There were 13 (19.1%) cases of GC with well differentiated, 20 (29.4%) cases with moderately differentiated, 35 (51.5%) cases with poorly differentiated. Furthermore, chronic superficial gastritis tissues (n=6) obtained from patients which underwent gastroscopic biopsy without GC were studied.

### Anticancer drugs

The anticancer drugs tested contained Taxol (TAX) (Taiji Co. Let., Sichuang, China), Cisplatin (CDDP) (Qilu Co. Let., Shandong, China), 5-Fluorouracil (5-FU) (Hualian Co. Let., Shanghai, China), Adriamycin (ADM) (Xinhua Co. Let., Shandong, China), and Mitomycin (MMC) (Huangshi Co. Let., Hubei, China). Each drug was diluted in a complete medium at 10-fold therapeutic peak plasma concentration as reported previously [8]. The complete medium consisted of RPMI 1640 (Gibco Gaithersburg, MD, USA) supplemented with 10% heat-inactivated calf serum (Gibco), 2 mM L-glutamine, and antibiotics (100 U/ml penicillin and 100 μg/ml streptomycin).

### MTT chemosensitivity assay

The *in vitro* chemosensitivity of fresh surgical specimens of GC was evaluated using the MTT assay as reported by Mossman, with some modifications [23]. The tissue specimens obtained during surgery were from patients who had given written informed consent. Resected specimens were stored in Hank’s balanced salt solution (Gibco Gaithersburg, MD, USA) that contained 100 IU penicillin, 100 μg streptomycin and 0.25 μg amphotericin B (all from Gibco) per ml. Single-cell suspensions were prepared enzymatically by incubating the specimens for 30 min in 0.5 mg/ml pronase, 0.2 mg/ml collagenase type І and 0.2 mg/ml DNase (all from Sigma). After 2 centrifugations (1000 r/min), the tumor cells were suspended in RPMI 1640 medium supplemented with 10% fetal bovine serum, diluted to 1×10^5^ cells/ml and 100 μl aliquots were plated into 96 well microplates (Gibco) to give approximately 10^4^ cells per well. The drug solutions were dissolved in RPMI 1640 and 100 μl aliquots were added to each well to give final concentrations of 10 μg/ml MMC, 50 μg/ml 5-FU, 25 μg/ml CDDP, 5 μg/ml TAX, or 4 μg/ml ADM. The control wells contained 100 μl of the cell suspension and 100 μl RPMI 1640 containing 10% FBS, and 200 μl RPMI with 10% FBS was used as a blank. The plates were incubated for 48 h at 37°C in a humidified atmosphere containing 95% air and 5% CO_2_. 20 μl mixture of 0.4% MTT (Sigma) and 0.1 M sodium succinate (each dissolved in 10 μl phosphate-buffered saline and filtered through a 0.45 μm membrane filter (Millipore, Bedford, MA, USA)), was then added and the plates were incubated for an additional 3 h at 37°C. After the final incubation, 150 μl dimethyl sulfoxide (Gibco) was added to each well to dissolve the MTT-formazan salt and the plates were mechanically shaken for 10 min on a mixer. The optical densities of each well were determined using a model SM-3 easy reader (Tianshi, Beijing, China) at 570 nm. The inhibition rates (IR) were calculated using the formula (1 – A/B) ×100%, where A and B represent the mean absorbance of the drug-treated and control wells, respectively. The effects were regarded as positive when IR values were ≥ 30%.

### hTERT assay

In situ hybridization (ISH) was carried out by using an hTERT ISH detection kit (produced by Wuhan Boster Biological echnology Ltd.). The antisense poly-oligonucleotide probe was digoxin-labeled. Formalin-fixed, paraffin-embedded samples were cut at 5 μm and adhered to poly-l-lysine treated slides. Samples were deparaffinized and rehydrated through a graded series of ethanol, and endogenous peroxidase was blocked using 3% hydrogen peroxide for 10 min. The slides were digested with pepsin at 37°C for 15–20 min. 20 μl of probe was hybridized to each slide for 16–20 h at 40°C. After hybridization, excess probe was removed by washing in 2×SSC at 37°C. Tissue sections were reblocked for 20 min with blocking reagent, and then the primary antibody (rabbit anti-digoxin antibody) was added for 60 min at 37°C. After washing with 0.5 M PBS three times at 5 min each, the slides were incubated with the secondary goat anti-rabbit immunoglobulin (IgG) antibody conjugated with biotin for 20 min at 37°C, then washed with 0.5 M PBS again as previously described. Samples were next incubated with SABC for 20 min at room temperature and rinsed with 0.5 M PBS for four times at 5 min each. The reaction products of peroxidase were visualized by incubation with chromogen diaminobenzidine for 15–20 min. Finally, the slides were counterstained for nuclei by haematoxylin stain. A negative control was prepared for each sample using a hybridization solution without probe. The positive signals of hTERT mRNA expression were stained with the color of brown-yellow located in cell plasma. The average percentage of positive cells was determined in at least 5 areas at ×400 and assigned to one of four categories: (−)-negative or equivocal staining; (+)-weak positive, cells were stained in 1-25%; (++)-middle positive, cells were stained in 25-50%; and (+++)-strong positive expression, cells were stained over 50%.

### Statistical analysis

Quantitative results were expressed as mean ± standard error of mean. Significant differences were determined by Fisher’s PLSD test or a Chi-square test. The associations analysis were tested with Spearman’s test for nonparametric correlation. A *P* value of less than 0.05 was considered to be statistically significant.

## Results

### Chemosensitivity of gastric cancer

In the study, a total of 68 GC tissue samples (lesions) were analyzed using the MTT assay. Sixty lesions were considered to be evaluable (success rate: 88.2%). After incubation with chemo-drugs for 48 h, the drug-sensitive cells lost their adherence abilities, increased their intracytoplasm vacuolus, and collapsed. The inhibition rates of tumor cells exposed to TAX, CDDP and 5-FU were significantly higher than those of ADM and MMC (*P*<0.05) (Table 1). The inhibition rate for TAX was equivalent to those for CDDP and 5-FU, but the estimated efficacy for TAX was higher than those for CDDP and 5-FU. Statistical analysis between the drug effects and the clinicopathological features showed a significant association between chemosensitivity to anticancer drugs and worse histological grades. Statistical differences of chemosensitivity to anticancer drugs in different tumor histological types were also observed. However, there were no significant differences of chemosensitivity in different TNM stages.

**Table 1:** Chemosensitivity of 60 cases GCs to five anticancer drugs

**Drugs**	**Chemosensitivity**			**Average inhibition rate (mean ± standard error)**
	**High**	**Middle**	**Low**	
TAX	6 (10%)	17 (28.3%)	37 (61.7%)	40.6% ± 9.9
CDDP	5 (8.3%)	16 (26.7%)	39 (65.0%)	38.4% ± 7.8
5-FU	5 (8.3%)	15 (25.0%)	40 (66.7%)	38.9% ± 9.2
ADM	2 (3.3%)	15 (25.0%)	43 (71.7%)	31.6% ± 8.5
MMC	1 (1.7%)	17 (28.3%)	42 (70.0%)	28.9% ± 9.8

Abbreviations: GC= gastric cancer, TAX =Taxol, CDDP=Cisplatin, 5-FU=5-Fluorouracil, ADM= Adriamycin, MMC= Mitomycin.

### Expression levels of hTERT mRNA

The examination of hTERT mRNA expression was done in GC tissues and corresponding normal gastric mucosa of 60 patients who were obtained successful MTT assay. In addition, 6 cases of chronic superficial gastritis mucosa from patients without GC also underwent this testing. The positive signal for hTERT mRNA expression was a brown-yellow stain located in the cell plasma. Carcinomas exhibited positive hTERT significantly more frequently than normal gastric mucosa tissues (*P*<0.05). Signals were observed in none of the six chronic superficial gastritis samples and 60 cases of normal gastric mucosa tissues, but in 90% (54 of 60) of the GC samples (Table 2). Statistical analysis of the relationships between hTERT mRNA expression and the clinicopathological features revealed a significant association between hTERT mRNA expression and a worse tumor differentiation (*P*=0.013). However, there were no significant associations between hTERT mRNA expression and the other clinicopathological findings in GC.

**Table 2:** hTERT mRNA expression in 60 cases GCs

**Tumor histopathologic types**	**Cases**	**hTERT mRNA expression**				
		**-**	**+**	**++**	**+++**	**Positive(%)**
Papillary adenocarcinoma	18	2	6	5	5	16 (88.9%)
Tubular adenocarcinoma	18	2	5	6	5	16 (88.9%)
Mucinous adenocarcinoma	12	1	3	4	4	11 (91.7%)
Signet-ring cell cancer	12	1	4	3	4	11 (91.7%)

Abbreviations: GC= gastric cancer, hTERT=human telomerase reverse transcriptase.

### Relationship between hTERT mRNA expression and chemosensitivity

Each drug was tested to assess the relationship between chemosensitivity to anticancer agents and hTERT mRNA expression in GC. A close relationship was seen between hTERT mRNA expression and 5-FU and ADM sensitivity (*P*<0.05) in GC. The sensitivities of TAX, CDDP, and MMC showed no association with the hTERT mRNA expression in GC.

## Discussions

We have successfully analyzed the chemosensitivity of GC samples to anticancer drugs using the MTT assay in 60 GC patients. Based on the overall results, GC cells were more susceptible to CDDP and 5-FU than to ADM and MMC, which coincided with previous reports [24,25]. TAX is a new drug used in chemotherapy for GC [26,27], to which GC cells were the most susceptible, and should be widely used in GC. However, tumor cells were lower susceptible to MMC than the other drugs, suggesting that MMC alone is not effective to treat most GCs [28]. In the present study, the response of tumor cells to the drugs was correlated with the histological type and grade of the tumor. Mucinous adenocarcinoma and signet-ring cell cancer were more sensitive to drugs than papillary adenocarcinoma and tubular adenocarcinoma. Cells with a poor histological grade also were more sensitive to drugs. This difference may be because the poorly differentiated cells usually have higher proliferative activity. It was well documented that great variety in the response of GC cells to chemotherapy drugs present, but the correlation between sensitivity and clinicopathological parameters was not clear. These data suggest that it is difficult to choose the appropriate chemotherapies for GC based on the clinicopathological parameters. Meanwhile, the efficacy of these drugs are less than 45%, and adverse effects cannot be ignored. Thus, chemosensitivity testing is essential to individualize chemotherapy, which could lead to the improvement in the quality of life for GC patients.

The hTERT induction and telomerase activation are crucial for transformed cells to stabilize their telomere length and maintain their replicative potential, whereas most normal human somatic cells lack telomerase activity due to the stringent repression of the hTERT gene [13,29,30]. Therefore, telomerase activity or hTERT assessment is a useful marker for diagnosis and prognosis of various types of cancers [13]. Consistent with previous reports [31,32], our data show that hTERT mRNA expression is higher in GC samples compared to their corresponding normal mucosa. Moreover, hTERT mRNA expression was associated with a worse histological grade in GC. Thus, it appears that upregulation of hTERT mRNA expression might be a predictor for GC with a worse grade.

A previous study showed that the drug resistance that developed against Paclitaxel, Docetaxel, Vincristine, and Doxorubicin in MCF-7 cells was independent of the expression of the hTERT gene and telomerase activity [33]. So we were interested in the relationship between hTERT mRNA upregulated expression and the drug-resistance in GC cells. Our data showed that upregulated hTERT mRNA expression was associated with 5-FU (*P*=0.006) and ADM (*P*=0.028) resistance in GC cells; so this finding suggested that hTERT mRNA expression may be related with some anticancer drugs resistance in GC patients, but its mechanism still need further study.

## Conclusions

The determination of sensitive chemotherapy drugs for gastric cancer (GC) is one of the greatest challenges of adjuvant therapy. In our study, we found that Taxol, Cisplatin and 5-Fluorouracil were more effective than Adriamycin and Mitomycin for GC, and the chemosensitivity to anticancer drugs was associated with tumor histological types and a worse tumor grade. Compared to normal gastric mucosa tissues, hTERT mRNA expression was significantly increased in GC, furthermore, its increased expression was related to a worse tumor differentiation and 5-Fluorouracil or Adriamycin drug-resistance in gastric cancer. Examinations of hTERT mRNA expression would have reference values on selection of chemotherapeutic drugs for gastric cancer patients.

## Competing interests

The authors declare that they have no competing interests.

## Authors’ contributions

LW and MG carried out all evaluation, and PFL and MG drafted the manuscript. YCC and YCY carried out the MTT chemosensitivity assay and hTERT assay. PFL and YCC collected the clinical data. LW, PFL and MG contributed to the conception and design of the study. All authors read and approved the final manuscript.
